# Supplementary material for: Metagenomics workflow for hybrid assembly, differential coverage binning, metatranscriptomics and pathway analysis (MUFFIN)
Source: PLoS Comput Biol. 2021 Feb 9;17(2):e1008716. doi: 10.1371/journal.pcbi.1008716 (PMC7899367; doi:10.1371/journal.pcbi.1008716)
Supplement: Attachment — Submitted filename: Reviewers Comment.docx [file pcbi.1008716.s011.docx]

# Responses to the Reviewers

**Comments to the Authors:**

**Please note here if the review is uploaded as an attachment.**

**Reviewer #1:**

**This paper describes MUFFIN, a workflow application for binning MAGs from shotgun metagenome data generated from both long and short-read sequencing platforms.**

**Overall, the paper is well written, reasonably thorough, and describes a workflow that is certainly needed. We are not aware of competing workflows that deal with long reads, and we feel that MUFFIN is poised to become a widely used tool!**

**Despite the overall quality of the paper, we do think there are a number of things that could be reworded, clarified, and revised. Please see below.**

We thank the reviewer for taking his time to review our manuscript. We will address the suggestions in detail below. More in general, we updated all tools and programs within MUFFIN and did a lot of extensive testing via conda, singularity, and docker to identify and remove bugs and issues. Mostly issues with conda and database management could now finally be resolved.

**Major comments --**

**While ONT sequencing is certainly a fast-advancing technology, in our limted experience it seems still to be mostly suitable for relatively low complexity communities, and this paper glosses over its current limitations. Perhaps we are wrong? If so, more references would be useful (than just the one). Regardless it would be good to qualify the statement here to make it clear that e.g. reference 6 is about hybrid assembly, and not purely about de novo long read sequencing in isolation. And/or it might be good to provide some minimal guidance (perhaps elsewhere in the paper) as to how many reads from which ONT platform would be useful.**

*Thanks for your suggestions, nanopore technology is quite a fast moving field. e.g. accuracy is currently around 98% and throughput per cell for metagenomic data around 20 Gigabases median (https://www.mdpi.com/2076-3417/10/21/7518).* *We added some more references for metagenomic sequencing via nanopore to the introduction as a few paper manuscripts already suggested using long-reads over short reads when performing metagenomic assembly. The change in the introduction: “Complete viral genomes can be already identified from environmental samples without any assembly step via nanopore-based sequencing [7]. Combined with a reduction in cost per gigabase [8] and an increase in data output, the technologies for sequencing long reads quickly became suitable for metagenomic analysis [9–12]”* (line 60)

*We also added the following section to step 1 of the assembly to highlight a bit how the user experimental data can be considered when executing MUFFIN - or e.g. if not enough long-read data could be produced: “Both approaches should be chosen based on the available amount of raw read data available to users. E.g., if more short read data is available, meta-spades should be the choice (long reads are “supplemental”). If more long-read data is available, e.g.,> 15 Gigabases (corresponds to a full MinION or GridION flow cell) flye should be used as the assembly approach.”* (line 124)

*Currently around one nanopore flow cell is enough to get lots of data out of a metagenomic sample (~20 Gbases) - We didn't wanted to dive to deep into numbers here as already new flowcells and higher throughput are already available (e.g. new chemistry should increase now the total Bases to around 30 Gigabases per flow cell), which would make statements here in the text more confusing to the reader and outdated after half a year or so.*

**line 108, "Binning is the most crucial step..." We are not sure that is true - certainly assembly would seem to be important, for example. Perhaps "a crucial step"?**

We thank the reviewer for this remark and agree that our wording was misleading as binning is not the sole crucial step in metagenomics study. We changed the sentence to: “*Binning is one of the most crucial steps during metagenomic analysis besides assembly*.”(line 129)

**A useful addition would be some discussion of why the tools chosen were chosen. Perhaps there was no reason other than that "they worked in our hands" (which is perfectly fine) but if there is more to add, please do! It might also be good to comment on whether the workflow is flexible enough to "swap in" different tools.**

We thank the reviewer for this comment and agree that justifying tools based solely on personal experience is not the optimum. In reality we selected the tools based on pre-existing benchmark performances (e.g. for assembly, binning etc.). We added the following section to the discussion chapter:“*MUFFIN utilizes a large number of tools to provide a comprehensive analysis of metagenomics samples. The associated tools were mainly chosen based on benchmark performance e.g., assembly [29,31,54–56], polishing [55], binning [15], annotation for pathways [49], taxonomic classification [46], however stability and workflow compatibility was also an important factor to consider. Due to the modular coding structure of nextflows DSL2 language, MUFFIN can quickly adapt towards better tools or improved versions if necessary, in the future*.“(line 236)

In addition, MUFFIN being developed in nextflow makes it a highly adaptive and flexible pipeline so we either way aim to add other options/tools here in the future. Other options are planned such as long-read only (v2), short read only (v2), additional analysis steps (v2/v3) as this is also a feature request within our institution and cooperating partners. We added our current plans to the Milestone section of the github to be more transparent (<https://github.com/RVanDamme/MUFFIN/projects>).

**Line 179, are there downsides to this re-assembly? Perhapos chimerism/loss of microdiversity? It would also be good to discuss why additional bins were able to be constructed on a second round, after being missed in the first round.**

To this day and to our knowledge, there are significant downsides and upsides found about doing re-assembly. Doing a reassembly in MUFFIN is therefore always an optional step and turned off by default.

Currently we retrieve the reads by mapping them to the whole assembly (not individual bins) and sort them back to each individual bin based on contig IDs. In this way we avoid that reads map more than once against bins. In addition the reassembly is done by unicycler running spades followed by rounds of polishing that should reduce even more the risk of chimerism. However chimerism is always an issue in metagenomic assembly, this is why some manual curation afterwards is always necessary after retrieving the bins.

Based on your comment we further checked the reassemblies and observed slight increase or decrease in quality (based on checkM scores). Also reassembly increases in some cases classification depth and decreases in others. We added the following part to the results to reflect this:

“*Moreover, an additional reassembly of bins has shown the capacity to increase the completeness and N50 while decreasing the contamination of some bins* [*[15]*](https://www.zotero.org/google-docs/?DAsYCi)*. Therefore, MUFFIN allows for an optional reassembly to improve the continuity of the MAGs further. This reassembly is performed by retrieving the reads belonging to one bin and doing an assembly with Unicycler (v0.4.7)* [*[38]*](https://www.zotero.org/google-docs/?YPx7qv)*. As each reassembly might improve or worsen each bin, this process is optional and therefore deactivated by default. Individual manual curation is necessary by the user to compare each bin before and after reassembly, as described by Uritskiy et. al.* [*[15]*](https://www.zotero.org/google-docs/?4nEKRq)*.*”(line 135)

and to the results of the reassembly:

”*Exemplarily, we investigated the impact of additional reassembly of each bin for five samples (Figure 3). The N50 was increased by an average of 6-7 fold across all samples. Twenty-six bins of the five samples had an N50 ranging between 1 to 3 Mbases. Reassembly of bins has shown the capacity to increase the completeness and N50 while decreasing the contamination of some bins* [*[15]*](https://www.zotero.org/google-docs/?lNRjif)*. This is in line with our samples as some bins benefit more from this step as others. In general, while we observed a general increase in N50 for most bins the genome quality based on checkM metrics (completeness, contamination) was slightly increasing or decreasing for individual bins.*”(line 209)

**Perhaps comment on why RNAseq reads are assembled instead of being mapped? What is being mapped to whom, in any case, line 137? Are the de novo metagenome assemblies/MAGs used at all here?**

We changed the line 137 to make the RNA-seq mapping for quantification more clearer. Indeed the mapping is done between the transcript and the RNA-seq reads to allow a quantification of the expression of the transcripts.
We added the following section to discuss the assembly of RNA-seq in the discussion chapter:”*MUFFIN executes a de novo assembly of the RNA-seq reads instead of a mapping of the reads against the MAGs to avoid bias and error during the mapping. Indeed, not all the DNA reads were assembled or binned and present in the last step (annotation). Thus we might miss transcripts on the sample level. In addition, for similar genes it’s impossible to know to which organism the reads should map to, by using metatranscripts and comparing the annotations of the metatranscripts to the annotation of the MAGs we avoid those issues.*”(line 253)

**Eggnog outputs a lot of information other than just KEGG... perhaps some mentions of these would be good? (GO terms, NOG terms, suggested taxonomy, etc)**

We thank the reviewer for the suggestion, this is indeed important information to some, we added this to the “Design and implementation” - “Step 3” part as follows: “ *the annotation by eggnog-mapper provides a wide array of annotation information such as the GO terms, the NOG terms, the BiGG reaction, CAZy, KEGG orthology and pathways.*”(line 156)

**Minor or easily resolved comments --**

**We suggest citing ATLAS and Sunbeam, which are short-read-focused workflows for MAG extraction.**

We thank the reviewer for the suggestion, we mentioned these tools and referenced them in the introduction section.

**Please make an archival copy of the version of MUFFIN used in this paper and provide the DOI; this can easily be done by connecting Zenodo to GitHub and making a new release on GitHub.**

Thanks for the suggestions we added a zenodo DOI (10.5281/zenodo.4296623) and MUFFIN release number (V1.0.3) to the manuscript.

**line 44, 'such' can be removed.**

We updated the manuscript.

**In Figure 1, what does the word "Differential" (in Differential Reads as an input) mean?**

These are reads that are solely used to improve binning results via “differential coverage binning”. By producing additional coverage information via other samples - therefore creating various additional “bam files” - the binner can achieve better bins. Differential coverage binning is mentioned in the manuscript and cited (Genome sequences of rare, uncultured bacteria obtained by differential coverage binning of multiple metagenomes. *Nat Biotechnol* 31, 533–538 (2013)). We added the following part to the figure: “*Differential reads are other read data sets that are solely used for "differential coverage binning" to improve the overall binning performance.*”(line 111)

**line 110, 'refine' should be plural**

We corrected the line 110.

**line 120, maybe suggest some additional tools beyond running MUFFIN again? mifaser, for example?**

We thanks the reviewer for the suggestion, we added some suggestion of additional tools: “ *These “unused” reads could be further analyzed by other tools such as Kraken2 [39], kaiju [40], or centrifuge [41] for read classification, “What the Phage” [42] to search for phages, mi-faser [43] for a functional annotation of the reads or even use these reads as a new input to run MUFFIN.* ”(line 146)

**line 126, sourmash v3.5 was released recently - perhaps upgrade past an alpha version?**

We thank the reviewer for the suggestion, we updated sourmash and the GTDB database used.

**line 182, "more _from_ this step"**

Changed.

**line 198, "whereas" should be rephrased - maybe refer to both technologies? Something like "MUFFIN takes advantage of short reads for abundance estimation and long reads for their ability to resolve repeats" or something .**

We thanked the reviewer for the suggestion, we rephrased the line to: “*MUFFIN utilizes the advantages of both sequencing technologies. Short-reads provide a better representation of low abundant species due to their higher coverage based on read count. Long-reads are utilized to resolve repeats for better genome continuity.*”(line 229)

**line 222, suggest removing both "all" words.**

Removed them.

**line 237, "Annotation" should not be capitalized.**

Changed.

**Figure 3A colors make it hard to see the most contaminated bins. Maybe invert the color scale? or choose the inferno palate instead of viridis.**

We changed the Figure to inferno coloring scheme.

**We suggest reporting not only the MAGs/length etc that MUFFIN recovered, but also how many reads weren't assembled/analyzed/didn't make it into the MAGs.**

This is a good suggestion to add, as for now it is possible to report those information manually but we will add this for the future version of MUFFIN as an automatically generated information for the user.

**Reviewer #2:**

**Long reads are more and more common for metagenomics. MUFFIN is a pipeline integrating short reads, long reads and RNA seq reads for functional annotation of metagenomes. It aggregates the results into Kegg pathways and produces nice html reports. It is great that the authors provided multiple executer systems. The installation instruction is clear, and the executor can be specified easily.**

We thank the reviewer for the appreciation of our review effort. More in general, we updated all tools and programs within MUFFIN and did a lot of extensive testing via conda, singularity, and docker to identify and remove bugs and issues. Mostly issues with conda and database management could now finally be resolved. We will address the suggestions in detail below.

**Major concerns:**

**1. Unable to run MUFFIN**

**I tried to run MUFFIN on a Linux cluster with different combinations of executors and backends without success. The executed commands together with the error logs are attached.**

We thank the reviewer for taking the time to try MUFFIN.

The issue with the conda environment “Can't locate LWP/Simple.pm” got solved in the latest commit, so you can git pull the release V1.0.3 and everything should be operational.

The error code of nextflow 143 when using slurm seems to come from memory issues and timeouts of slurm. We optimized the pipeline to have a better suggestion of the RAM but in case the error persists, trying to use more memory should solve the issue. For this we added a read the doc webpage for MUFFIN (https://rvandamme.github.io/MUFFIN_Documentation) to add frequently asked questions and some insights on how to quickly adjust some parameters that might be unique to your own cluster environment.

In annotate_muffin.sh you are loading a Singularity module to run MUFFIN but uses the “-profile docker”. We recommend you use the singularity profile instead as we pre configured each process with a individual container.

**2. More documentation to customize the cluster execution.**

**It is impossible to make a tool that can be run on any cluster system. The authors did a good job by providing solutions for slurm and google could. However, if they claim that MUFFIN can be executed on other systems (L160) they should provide profiles or document how users can create execution profiles for their clusters. It would also be helpful to explain how to configure the profiles.**

We thank the reviewer for the suggestion, we are working on a readthedoc for the MUFFIN pipeline available at <https://rvandamme.github.io/MUFFIN_Documentation/> . We add a troubleshooting section that we extend based on community feedback, a tutorial for how to setup new configuration profiles and how to change parameters in existing profile is also available for people who want to try this without creating a github issue.

**3. Multiple samples.**

**It is not completely clear how a user can run MUFFIN with multiple samples. Does the user need to run MUFFIN on each sample separately and define the optional other samples for differential binning? Is there no easier way to run MUFFIN on a set of samples? How can a user compare different samples? The main output of MUFFINs is the quantification of KEGG pathways based on MAGs (and RNA seq data) but the MAGs are sample-specific and not directly comparable.**

We thank the reviewer for this comment and agree that comparing different samples is currently impossible in this pipeline. MUFFIN was not developed to do comparative metagenomics but retrieve MAGs and provide key information about the functions of the MAGs. Due to the differential coverage binning and therefore multiple different read inputs, we currently don’t have an elegant solution for multiple sample inputs and differential binning.

As for the differential binning, when you run a sample, you can provide supplementary samples that will be used in the differential binning (e.g. own read sets or something from NCBI/ENA). Those extra samples improve the binning by providing similar data to the one analyzed but with variability in the population and abundance of the population, allowing Metabat2 and Concoct to improve their binning. Unfortunately, the pipeline was not intended to run different samples at once yet, but based on user feedback we might implement this in the future.

The quantification of the KEGG orthologs found by de novo transcript assembly is only an additional file in the result and doesn’t aim to be a comparative file between samples. But serve to inform which of the transcripts were the most/least expressed in the sample, allowing to infer what is the “principal” function at the time of sampling in that specific sample. MUFFIN currently doesn’t quantify but highlights the gene expressed.

**4. RNA-seq:**

**If I understand correctly the RNAseq data comes from a microbial community, not from a single genome. In this case, the term “metatranscriptome” would be more appropriate.**

We thank the reviewer for the suggestion, we updated the term transcriptome to metatranscriptome.

**Trinity was originally not designed for meta-transcriptomes but works relatively good (10.1186/2049-2618-2-39). Could you discuss the challenges of assembly for metatranscriptomes and why you used genome-independent transcriptome assembly if you have MAGs available?**

We added a section to discuss this the assembly of RNA-seq in the discussion chapter: “*MUFFIN executes a de novo assembly of the RNA-seq reads instead of a mapping of the reads against the MAGs to avoid bias and error during the mapping. Indeed, not all the DNA reads were assembled or binned and present in the last step (annotation). Thus we might miss transcripts on the sample level. In addition, for similar genes it’s impossible to know to which organism the reads should map to, by using metatranscripts and comparing the annotations of the metatranscripts to the annotation of the MAGs we avoid those issues.*”(line 253)

**5. Pathway aggregation.**

**MUFFIN provides nice html reports with of the KEGG pathways and the genes present in the MAGs and or the RNA seq data. Unfortunately, the gene presence (at least in the provided subset results) is relatively sparse. It is therefore difficult to interpret and compare the data. Could you provide a pathway coverage information as numerical values? It would also be interesting to see pathways of the 20 samples from the bioreactor project as a figure.**

We thank the reviewer for the suggestion, we added pathway coverage, as well as other types of output for the final results, as part of the milestone v3.

**6. Bioreactor example:**

**a. Did the re-assembly reduce N50 for some of the bins?**

You can see in figure 3B that in most cases the N50 was increased after the reassembly. On a more general view, the N50 improvement heavily depends on sample type sequencing size (Gb) and microbial composition (e.g. more diversity lots of low abundant organisms etc.)

As we discussed with Reviewer 1 and discuss in point 6.b., to this day and to our knowledge, there are significant up- and downsides found for doing re-assembly on all of the bins.

Therefore, doing a reassembly in MUFFIN is only an optional and experimental step (deactivated by default), you can run the pipeline with and without, as the pipeline keep every intermediary file you can even do a further comparison between the two methods. As the reassembly is done based on the bins found, no microdiversity should be lost nor chimerism should occur. The reassembly step retrieves the reads mapping to a bin then re-assembles them, thus no “foreign” reads should be present during the reassembly. In addition, the reassembly is done by unicycler running spades followed by rounds of polishing that should reduce even more the risk of chimerism. Still, manual curation by hand is still recommended to decide if reassembled bins are trustworthy.

**b. Did it change the quality?**

The quality (based on checkM completeness and contamination) varied greatly based on each bin (for some quality went up for some quality went down). However, this is purely based on checkM metrics. For the 5 samples shown in the paper we increased the N50 (see Figure 3B), for the classification by sourmash we obtained in some cases a better taxonomic depth for classification (e.g. down to species taxon) and in some cases a decrease.
All in all, if we base our assessment of quality on checkM (quality control based by a set of known markers) most of the time, it is not worth it to do a reassembly purely based on contamination and completeness values. Based on different parameters (sourmash classification, checkM quality, N50), it can achieve better MAG quality. This is why the option is optional for now. We extended the section to make this more clear:

“*Exemplarily, we investigated the impact of additional reassembly of each bin for five samples (Figure 3). The N50 was increased by an average of 6-7 fold across all samples. Twenty-six bins of the five samples had an N50 ranging between 1 to 3 Mbases. Reassembly of bins has shown the capacity to increase the completeness and N50 while decreasing the contamination of some bins* [*[15]*](https://www.zotero.org/google-docs/?A7o8lR)*. This is in line with our samples as some bins benefit more from this step as others. In general, while we observed a general increase in N50 for most bins the genome quality based on checkM metrics (completeness, contamination) was slightly increasing or decreasing for individual bins.*”(line 209)

**c. Why is the re-assembly only performed for 5 samples?**

The reassembly is an optional step that has heavy computer requirements (executing hundreds of unicycler runs) which might not improve the quality of all the bins (based on checkM). For this reason we just picked the first five samples and did not dive deeper into adding more and more samples as N50 improvement was the main advantage but this might be heavily affected by the sample type and we just want to highlight this example. We do not have any reason to believe that this might change for the other 15 samples. We aim to provide the opportunity for the user to execute a reassembly if they deem it necessary to do one (e.g. if genome continuity is their main goal), we do not promote or glorify the use of the reassembly.

**Minor**

**• There is no quality control implanted for long reads (except length filtering). Doesn’t it make sense to remove reads that come from contaminants or have low quality?**

The Flye assembly software recommends the use of raw nanopore reads as the software was developed for this type of reads, It was thus deemed not required to execute a quality control beforehand. Adapter and barcode trimming is also included in the guppy base caller. As MUFFIN aims to be open to a wide range of topics within metagenomic the contaminant list is uncertain. We plan to integrate the possibility to provide contaminant sequences to discard in a future update of MUFFIN. Especially removal of eukaryotic cells is a requested feature.

**• The Author summary paragraph is not the same as author contribution.**

We thank the reviewer for highlighting this error, we corrected it.

**• Novelty: L71: It seems as if the authors make the indirect claim that MUFFIN the first pipeline for hybrid assembly, is this true?**

According to our knowledge, there is no other “hybrid metagenomic pipeline” available online that does what MUFFIN provides (assembly, classify, annotate). If we only consider the hybrid assembly part of the pipeline, there are of course programs already available such as unicycler (mainly culture based but nowadays good for metagenomics) or HASLR (<https://www.sciencedirect.com/science/article/pii/S2589004220305770>).

**• The sentence L23-25 in the abstract is difficult to read. propose reformulation: “and can be further used for quantification and annotation by providing RNA-Seq data (optionally).”**

We thank the reviewer for the suggestion, we rephrased (line 25)

**• Is it possible to assemble with long reads only or does one need to define always short reads for the polishing?**

Long reads only assembly is planned in Milestone v2 and currently not supported as we need to adjust the binners first and their performance with long reads only.

**• L98: Are Pac Bio long also supported?**

Long reads only and hybrid assembly using Pacbio is planned in Milestone v2. Currently, the long-read assembler assumes nanopore raw read data. However, one could supply long reads of PacBio via the nanopore flag and use the meta spades assembler for now.

**• L127: Is it possible to update the GTDB ?**

Yes, the GTDB was updated alongside sourmash in MUFFIN release v1.0.3. If a major release of the GTDB comes out, and after testing the new release, we will update MUFFIN. An advanced user can also edit MUFFIN to use another database (GTDB or other) formatted in the right version for sourmash and use it.

**• L129: The sentence "GTDB substantially improved overall downstream results40" is misleading. One would assume that the sentence is about the downstream results of MUFFIN. But then the authors should explain which downstream results and how instead citing ref40.**

Thanks for pointing that out, we rephrased the sentence to make our statement more clear: “*Moreover, the coherent taxonomic classifications and more accurate taxonomic boundaries (e.g., for class, genus, etc.) proposed by GTDB substantially increases the general classification accuracy*”(line155)

**• The authors look ahead on potential future version conflicts. However, I don't fully understand the solution they propose. How can a user make sure that they pick the correct version of nextflow or MUFFIN? Isn't there another way where it's not up to the user to read the release notes to exclude version conflicts e.g. a conda package providing the tested version of MUFFIN and nextflow?**

This issue was related to the so-called "DSL=2" syntax of nextflow as this was still a beta-feature but highly desirable to use for any extensive workflow such as MUFFIN. DSL2 left the beta stage and is now completely integrated (This happened after submitting this manuscript). So we don't expect these issues anymore. Furthermore, MUFFIN checks the nextflow version to ensure that the user is not using a very old nextflow version that might be incompatible. We updated the section related to the version control to: “*Each MUFFIN release specifies the Nextflow version it was tested on but any version of MUFFIN V1.0.2+ will work with nextflow version 20.07+.*” (line 182)

There is no need to install a dedicated “conda package” as nextflow has a build in version control via “-r” to call any specific version of MUFFIN directly (this is explained in the readme section).

**• It's confusing that the '-profile' command-line argument has only one leading hyphen which is inconsistent with the rest of the CLI.**

We completely agree that it is confusing, but unfortunately we cannot change it. The -profile command line is a nextflow parameter to manage various execution profiles individually (similar to the nextflow parameter to resume an workflow execution by adding -resume) so it is separate from the pipeline parameters of MUFFIN (which we can edit). So basically: one minus nextflow related parameters; two minus workflow related parameters. An error code for --profile has been implemented to tell the user to remove a “-”.

Furthermore MUFFIN allows for a “parameter input file” which might greatly simplify usability. While not addressing the --profile issue all the inputs have “one -”; the command would look like this:

nextflow run RVanDamme/MUFFIN -params-file MUFFIN_params.yml -profile local,singularity

We added this optional input possibility to our documentation (https://rvandamme.github.io/MUFFIN_Documentation/usage/usage/)

**Reviewer #3:**

**The authors devised a particular metagenomic analysis workflow and applied it to 20 samples of public data. The workflow is composed of popular software tools and the orchestration software. The manuscript is focused on presenting this particular workflow, yet without giving any justification for the choices or discussion of alternatives. The description of the analysed samples is superficial to the point of being non informative.**

**Thus I struggled to apply criteria for publication in PLOS Computational Biology here:**

**• Originality - seems to be mostly related to hybrid assembly from short and long reads, which is commonly done when such data exists, which on itself is not very common.**

We thank the reviewer for taking the time to review our manuscript. MUFFIN aims to do automatically multiple time and resources consuming task such as hybrid assembly, differential binning, bin refinement, reassembly, classification by GTDB and checkM, annotation, de novo metatranscriptomics quantification and annotation (if provided) and the production of simple graphical output of the pathways and orthologs present in the sample. Therefore the above statement about MUFFIN just being an “hybrid assembler” is completely misleading and only describes the first process of many (see also Figure 1). The statement that this is not common strikes me as superficial, especially with regard to other very widely used and cited hybrid tools such as Unicycler. Especially long reads have become more and more interesting for scientists in recent years, as this technology has become more accessible to many laboratories via the nanopore sequencer following lots of reports of novel completed genomes from metagenomic sequencing. (See also Reviewer 1 remarks on MUFFIN)

**• Innovation - all the components are public 3rd party tools, thus it seems to be mostly related to a particular configuration of the pipeline.**

Automated bioinformatic pipeline is the next step in bioinformatic as this allows a biologist with basic training in computing to install tools and run large and or complex analysis. This aspect is also reflected via <https://nf-co.re/> and their efforts to provide complex analysis workflows easily to the scientific community. Using pipelines also provides multiple advantages such as a high reproducibility, total version control while reducing hands on time down from weeks to a few days via automation and parallelization.

MUFFIN is indeed composed of several 3rd party tools, and an environment with predefined parameters, a complete management of the intermediate files, the possibility to run all those 3rd party tools on various computer, cluster and cloud systems. In addition MUFFIN provides parsers and scripts to summarize, illustrate and highlight the results produced by the pipeline (e.g. classification, kegg annotation). To our knowledge there are no 3rd party tools that produce files containing the list of pathways and orthologs annotated on bins and produce a visual representation of the orthologs present or not in the said pathways across hundreds of bins.

**• High importance to researchers in the field - likely not.**

Combining long reads for genome continuity and “rather cheap” short reads for accuracy and coverage information is a straightforward advantage in metagenomics compared to doing each of them individually. Hybrid approaches in general are widely used in the scientific community (see unicycler for example). Therefore we do not agree with this statement. Furthermore MUFFIN plans to implement long and short read “only” options to extend its audience and capabilities in the future.

**• Significant biological and/or methodological insight - no.**

To our knowledge there is no workflow available for the described steps that MUFFIN provides. As mentioned in the previous point, hybrid assembly provides deeper assembly allowing better binning and further downstream analysis. MUFFIN also provides a graphical synthesized version of the annotation of the bins using KEGG pathways and orthologs. This visual does not bring new information per se as it is based on the user input data, but brings new perspective in an easy and compact way of conveying the KEGG pathways information.

**• Rigorous methodology - alternatives exist for each step, yet not evaluated.**

We agree with the reviewer that multiple alternatives exist for most of the steps present in the MUFFIN pipeline and it was our mistake on not pointing out clearer how we choose each tool. In short we evaluated previous benchmarks on each tool to decide which approach to take. However as MUFFIN is written in nextflow DSL2 we can and will easily add and exchange tools if they perform better in the future due to the modular structure as the bioinformatic field is usually fast moving. We added the following section to provide more clarity:

**“***MUFFIN utilizes a large number of tools to provide a comprehensive analysis of metagenomics samples. The associated tools were mainly chosen based on benchmark performance e.g., assembly [29,31,54–56], polishing [55], binning [15], annotation for pathways [49], taxonomic classification [46], however stability and workflow compatibility was also an important factor to consider. Due to the modular coding structure of nextflows DSL2 language, MUFFIN can quickly adapt towards better tools or improved versions if necessary, in the future.***”**(line 246)

Furthermore, the analysed samples present here are used to put emphasis on the different options and results MUFFIN provides when analysing metagenomes.It was not part of our scope for the Software description to describe biogas reactor samples.

**• Substantial evidence for its conclusions - no conclusions.**

Indeed there is no conclusion in this paper as we present a new pipeline for metagenomic based on hybrid assembly. We followed the software submission guidelines (<https://journals.plos.org/ploscompbiol/s/submission-guidelines#loc-software-submissions>).

The software should bring new biological insight, which we do by providing structured results, visualisation of the pathways present in the sample and bins and if RNA-seq data is provided the expressed orthologs in the pathways. The software should also be widely adopted or promising a wide adoption, based on the interest brought to our preprint we also fulfill this requirement. No conclusions chapter where provided in the article but we still demonstrate the use of our pipeline and every step of it, with the accompanying documentation.

**It reads as a part of documentation rather then a research article.**

It is part of the “software section” to describe open source software.

**Apologies for not being able to be more positive.**

Thank you for taking your time in reviewing our manuscript.

**Have all data underlying the figures and results presented in the manuscript been provided?**

Large-scale datasets should be made available via a public repository as described in the *PLOS Computational Biology* [data availability policy](http://journals.plos.org/ploscompbiol/s/data-availability), and numerical data that underlies graphs or summary statistics should be provided in spreadsheet form as supporting information.

Reviewer #1: Yes

Reviewer #2: **No:** could you provide the results from the 20 sample bioreactor or at least the important part of it?

All the bins from the 20 samples are now available at <https://osf.io/m5czv/>

Reviewer #3: **No:** missed the data for Figure 3.

All the bins from the 20 samples are now available at <https://osf.io/m5czv/>

PLOS authors have the option to publish the peer review history of their article ([what does this mean?](https://journals.plos.org/ploscompbiol/s/editorial-and-peer-review-process#loc-peer-review-history)). If published, this will include your full peer review and any attached files.
